# Accumulation of 2-methylcitrate induces metabolic imbalance in *Bacillus thuringiensis*, revealing a detoxification strategy mediated by an internal promoter

**DOI:** 10.3389/fmicb.2026.1675856

**Published:** 2026-01-28

**Authors:** Cuiying Du, Lanteng Zheng, Ke Fu, Rui Wang, Zhuofan Liu, Fengxian Liang, Chenyi Zeng, Xuanmingyue Zhou, Tingting Yang, Yujun Dai, Bingyue Xin, Cao Zheng

**Affiliations:** 1 Hubei Province Research Center of Engineering Technology for Utilization of Botanical Functional Ingredients & Hubei Key Laboratory of Resource Utilization and Quality Control of Characteristic Crops, College of Life Science and Technology, Hubei Engineering University, Xiaogan, Hubei, China; 2 Anhui Province Key Laboratory of Pollutant Sensitive Materials and Environmental Remediation, College of Life Sciences, Huaibei Normal University, Huaibei, Anhui, China

**Keywords:** 2-Methylcitrate, 2-methylcitrate cycle, *B. thuringiensis*, metabolic imbalance, PrpD

## Abstract

Propionic acid is a common food preservative, but many microbes, including the important biocontrol agent *Bacillus thuringiensis*, can metabolize it via the 2-methylcitrate cycle. However, the accumulation of cycle intermediates, such as 2-methylcitrate, can be toxic, and the overall physiological effects of this toxicity on *B. thuringiensis* are unclear. In this study, we investigated the toxic effects of 2-methylcitrate on *B. thuringiensis* and its corresponding cellular responses by characterizing the *prpD* deletion mutant Δ*prpD*, which lacks the 2-methylcitrate dehydratase. We found that the accumulation of 2-methylcitrate in the Δ*prpD* mutant led to a sharp decline in biomass, extensive cell lysis and death during the stationary phase. Comparative transcriptomic analysis revealed that this toxicity is associated with severe overall metabolic imbalance, characterized by a significant transcriptional dichotomy: concerted downregulation of nearly all glycolytic pathway genes and simultaneous upregulation of TCA cycle genes. This transcriptional decoupling of central carbon metabolism is the root cause of the observed lethal phenotype. Furthermore, we identified and characterized an internal promoter located within the *prp* operon that specifically drives *prpD* expression. This internal promoter rapidly and efficiently clears toxic intermediates, representing a complex regulatory adaptation mechanism to combat the harmful effects of propionic acid metabolism. Our findings provide a comprehensive transcriptional view of the toxicity of 2-methylcitrate and reveal a unique bacterial metabolic detoxification strategy, highlighting the value of PrpD as a potential anti-bacterial target.

## Introduction

**Figure 1:**
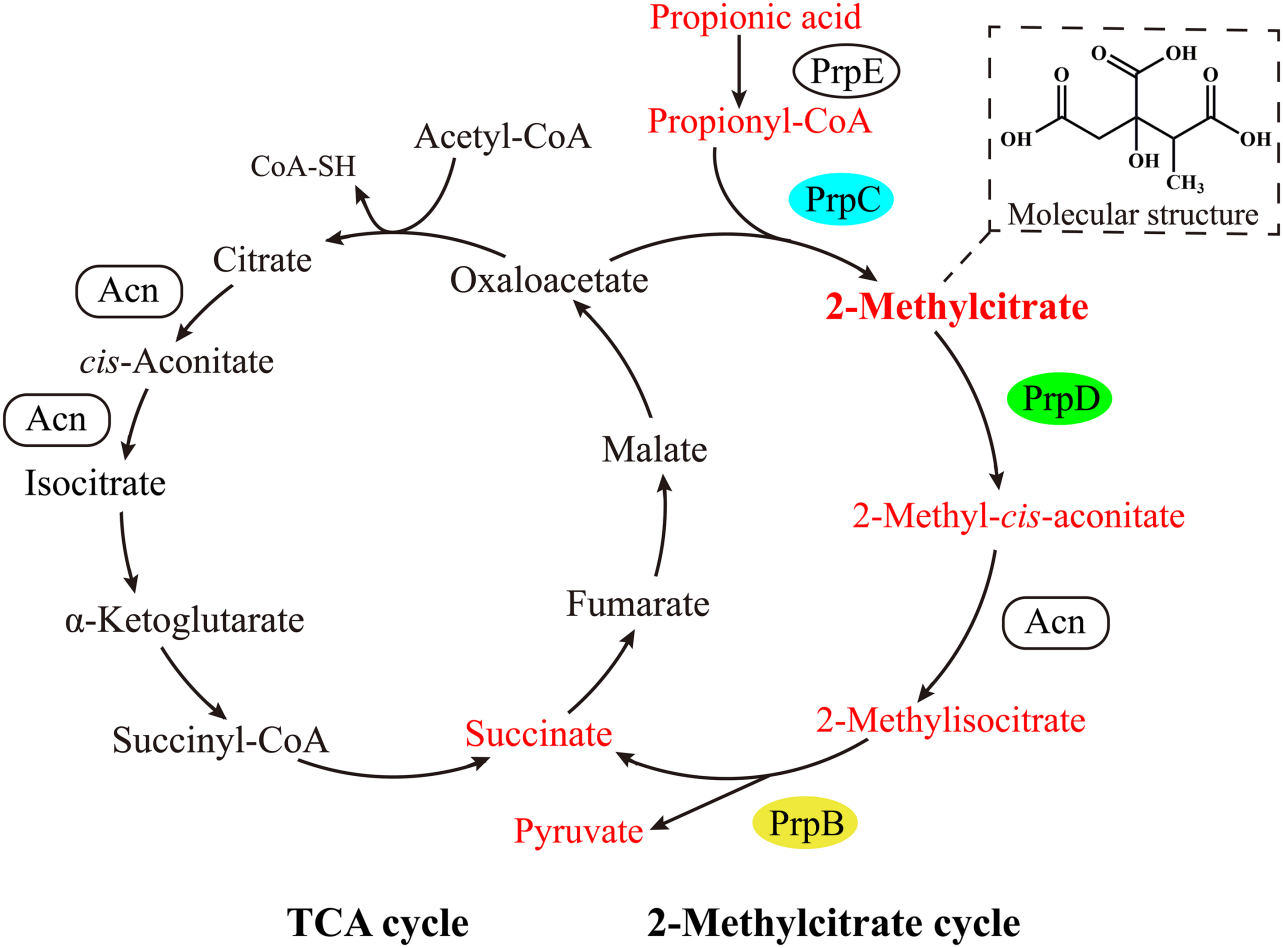
The 2-methylcitrate cycle in bacterial propionic acid catabolism. The pathway is structurally and enzymatically similar to the tricarboxylic acid (TCA) cycle. The key enzymes specific to the 2-methylcitrate cycle are 2-methylcitrate synthase (PrpC), 2-methylcitrate dehydratase (PrpD), and 2-methylisocitrate lyase (PrpB), highlighted in cyan, green, and yellow, respectively. In many bacteria, including *B. thuringiensis*, the genes encoding these enzymes (*prpC*, *prpD*, and *prpB*) are located in the same *prp* operon. The chemical structure of the intermediate 2-methylcitrate is shown in the upper right corner.

Propionic acid is a short-chain fatty acid widely found in natural environments. It is commonly used as a food preservative because it inhibits microbial growth and prevents food spoilage (Collograi et al., 2022; Ranaei et al., 2020). Despite its inhibitory effects, many bacteria and fungi have evolved metabolic pathways to use propionic acid as a carbon and energy source (Vestal and Perry, 1969; Pettinato et al., 2025). The most common of these is the 2-methylcitrate cycle (Textor et al., 1997; Uchiyama et al., 1982). Before entering this cycle, propionic acid must be activated to propionyl-CoA by propionyl-CoA synthetase (PrpE) (Horswill and Escalante-Semerena, 1999) (Figure 1). Subsequently, 2-methylcitrate synthase (PrpC) catalyzes the condensation of propionyl-CoA and oxaloacetate to produce 2-methylcitrate (Reddick et al., 2017) (Figure 1). This intermediate is then isomerized to 2-methylisocitrate via a series of dehydration and hydration reactions catalyzed by 2-methylcitrate dehydratase (PrpD) and aconitase (Brock et al., 2002) (Figure 1). Finally, 2-methylisocitrate lyase (PrpB) cleaves 2-methylisocitrate into succinate and pyruvate, both of which can enter the central metabolic pathways to support microbial growth (Grimek et al., 2003) (Figure 1).

Although the 2-methylcitrate cycle can catabolize propionic acid, its intermediates can have adverse effects on cell physiology (Dolan et al., 2018). For instance, high levels of propionyl-CoA can severely inhibit CoA-dependent enzymes, such as pyruvate dehydrogenase and succinyl-CoA synthetase in *Aspergillus nidulans* and *A. fumigatus*, leading to growth retardation (Maerker et al., 2005; Brock and Buckel, 2004). In *Salmonella enterica*, the accumulation of 2-methylcitrate inhibits fructose-1,6-bisphosphatase (a key enzyme in the gluconeogenesis pathway), thereby hindering its growth (Rocco and Escalante-Semerena, 2010). Moreover, the accumulation of 2-methylcitrate is a biochemical marker of propionic acidemia and other congenital propionate metabolism defects in humans (Amaral et al., 2016; Zhang et al., 2023). This suggests that propionic acid detoxification is a multi-step process that requiring fine-tuning to prevent the accumulation of toxic intermediates.

*Bacillus thuringiensis* (Bt) is a Gram-positive, spore-forming bacterium best known for producing insecticidal crystal proteins (ICPs), making it one of the most widely used biological pesticide in agriculture (Peng et al., 2019; Shi and Sun, 2025). The metabolic state of *B. thuringiensis* plays a crucial role in regulating key physiological processes, including sporulation and ICP synthesis (Wang et al., 2013). The 2-methylcitrate cycle, in particular, with its intermediates, is thought to be involved in the regulation of sporulation (Zheng et al., 2020). Our previous work has shown that deletion of the *prpD* gene, which encodes a key 2-methylcitrate dehydratase, leads to elevated 2-methylcitrate levels and adversely affects the growth of *B. thuringiensis*. Although studies have shown that 2-methylcitrate can inhibit specific enzymes in central carbon metabolism, our overall understanding of its effects on multiple metabolic pathways remains incomplete.

In this study, we demonstrate that the deletion of the *prpD* gene in *B. thuringiensis* leads to cell lysis and death. Comparative transcriptomic analysis revealed that this phenotype is associated with severe metabolic imbalance, characterized by opposing changes in the transcriptional levels of the glycolytic pathway and the TCA cycle. Furthermore, we discovered a novel detoxification strategy in *B. thuringiensis*: an independent promoter within the *prp* operon specifically upregulates *prpD* expression, enabling cells to rapidly clear the toxic 2-methylcitrate.

## Materials and methods

### Bacterial strains, plasmids, and culture conditions

The bacterial strains, plasmids, and primers used in this study are listed in Table 1 and Supplementary Table S1, respectively. *B. thuringiensis* BMB171, an acrystalliferous mutant strain with high transformation efficiency (He et al., 2010), was used as the parent strain. Unless otherwise specified, all Bt strains were cultured at 28 °C and 200 rpm in GYS medium (g/L: glucose, 1; yeast extract, 2; K_2_HPO_4_·3H_2_O, 0.655; (NH_4_)_2_SO_4_, 2; MgSO_4_·7H_2_O, 0.041; MnSO_4_·H_2_O, 0.0378; CaCl_2_, 0.08). When required, the final concentration of erythromycin was 25 μg/mL, and the concentration of vitamin B_12_ supplementation was 10–50 μg/mL. For routine cloning, *Escherichia coli* DH5α was cultured at 37 °C on lysogeny broth (LB) medium or LB agar plates, supplemented with ampicillin (100 μg/mL) as needed.

**Table 1:** Bacterial strains and plasmids used in this study.

Bacteria or plasmids	Relevant characteristics	Origins
Bacterial strains		
*Escherichia coli* DH5α	RecA1 endA1 gyrA96 thi hsdR17(r_k_^−^ m_k_^+^) relA1 supE44 Φ80ΔlacZΔM15Δ(lacZYA-argF)U169	Beijing TransGen Biotech Co., Ltd
BMB171	*Bacillus thuringiensis* strain BMB171; an acrystalliferous mutant strain; high transformation frequency	He et al. (2010)
Δ*prpD*	*prpD* mutant of BMB171	Zheng et al. (2020)
CΔ*prpD*	The *prpD* complementary strain where *prpD* is expressed in *trans* in the Δ*prpD* mutant	This work
BMB171/pHT1K-*P_prpD_*-*lacZ*	BMB171 containing pHT1K-*P_prpD_*-*lacZ* plasmid	This work
BMB171/pHT1K-*P_prp_*-*lacZ*	BMB171 containing pHT1K-*P_prp_*-*lacZ* plasmid	Zheng et al. (2020)
BMB171/pHT1K- *lacZ*	BMB171 containing pHT1K-*P_prpD_*-*lacZ* plasmid	This work
Plasmids		
pHT1K-*lacZ*	pHT1K plasmid harboring the promoterless *lacZ* gene	Zheng et al. (2015)
pHT1K-*P_prp_*-*lacZ*	*lacZ* with the promoter of *prpC* (*prp* operon) in NcoI and BamHI sites of pHT1K	Zheng et al. (2020)
pHT1K-*P_prpD_*-*lacZ*	*lacZ* with the promoter of *prpD* in NcoI and BamHI sites of pHT1K	This work
pRP1028	*Bacillus thuringiensis*-*Escherichia coli* shuttle plasmid; Spc^R^; containing *turbo-rfp* gene and an I-SceI recognition site	Zheng et al. (2015)
pSS4332	*Bacillus thuringiensis*-*Escherichia coli* shuttle plasmid; Km^R^; containing *gfp* and I-SceI restriction enzyme-encoding gene	Zheng et al. (2015)
pSS1827	The helper plasmid for conjugative transfer; Amp^R^	Zheng et al. (2015)
pRP1028-C*prpD*	The *prpD* complementary vector, which was generated by inserting the *prpD* gene with its flanking homologous arms into the pRP1028 plasmid	This work

### Construction of the *prpD* complementary strain

To construct a *prpD* complementary strain, the *prpD* gene and its upstream and downstream flanking regions were first amplified from the BMB171 genomic DNA using PCR. The amplification product was then cloned into the pRP1028 vector to construct a complementary plasmid. Finally, this plasmid was introduced into the recipient strain Δ*prpD*. The resulting complementary strain, named CΔ*prpD*, was obtained using an I-SceI-mediated markerless gene editing protocol (Zheng et al., 2015).

### Cell dry weight determination

Strains BMB171, Δ*prpD*, CΔ*prpD* and Δ*prpCDB* were cultured as described above. 20 mL of culture medium was collected every 2 h. The culture medium was centrifuged at 12,000 × g for 10 min to collect the cells. The resulting cell pellet was washed once with distilled water and dried in a 100 °C oven to constant weight for cell dry weight determination.

### Transmission electron microscope analysis

Strains BMB171 and Δ*prpD* were cultured in GYS medium at 28 °C. At specified time points, 4 mL cell samples were harvested by centrifugation. The cell pellet was fixed overnight at 4 °C with 2.5% glutaraldehyde. Sample preparation, including ultrathin sectioning and staining, was performed as previously described (Zheng et al., 2020). Sections were observed using a Hitachi H-7650 transmission electron microscope (Hitachi, Japan).

### RNA isolation and quantitative transcriptomics (RNA-seq)

Strains BMB171 and Δ*prpD* were cultured in GYS medium for 12 h. 24 mL aliquots of each culture were harvested, and total RNA was isolated using TRIzol reagent (Invitrogen, Carlsbad, CA, USA) according to the manufacturer’s protocol. RNA quality was assessed by 1% agarose gel electrophoresis, and RNA concentration was quantified using a NanoDrop spectrophotometer (Thermo Scientific, USA). RNA integrity was verified using an RNA 6000 Pico LabChip on an Agilent 2,100 Bioanalyzer (Agilent, Santa Clara, CA, USA). Ribosomal RNA removal, cDNA library construction, and Illumina sequencing were performed by a commercial service provider as described previously (Zheng et al., 2020; Yu et al., 2022). Two biological replicates were conducted on each sample.

### RNA-seq data analysis

The quality of the raw sequence data was assessed using FastQC. Adapters and low-quality sequences were trimmed using PRINSEQ. The resulting clean reads were aligned to the *B. thuringiensis* BMB171 reference genome (GenBank accession: CP001907.1) using Bowtie2. Gene expression levels were quantified as Reads Per Kilobase of transcript per Million mapped reads (RPKM). Differentially expressed genes (DEGs) between the Δ*prpD* and BMB171 strains were identified using the DEGseq package with the MARS algorithm. Genes with a false discovery rate (FDR) < 0.001 and an absolute log_2_(Fold Change) > 1.0 were considered significantly differentially expressed. The RNA-seq data have been submitted to NCBI Gene Expression Omnibus (GEO) database under the accession number GSE140317.

### Construction of the transcriptional fusion plasmid

A 250 bp DNA fragment corresponding to the intergenic region between the *prpC* and *prpD* genes was amplified from the BMB171 genomic DNA using the primer pair *P_prpD_*-*lacZ*-F/*P_prpD_*-*lacZ*-R (Supplementary Table S1). The PCR product was double-digested with NcoI and BamHI and ligated to the corresponding sites in the *lacZ*-reporter shuttle plasmid pHT1K-*lacZ* (Zheng et al., 2015). The ligation product was transformed into *E. coli* DH5α, and the resulting plasmid, designated pHT1K-*P_prpD_*-*lacZ* (Table 1), was verified by DNA sequencing. The plasmid was subsequently introduced into *B. thuringiensis* BMB171 by electroporation. Transformants were selected on LB agar plates containing 25 μg/mL erythromycin. The reporter plasmid pHT1K-*P_prp_*-*lacZ* for monitoring the transcriptional activity of the entire *prp* operon has been described previously (Zheng et al., 2020).

### *β*-Galactosidase activity assay

BMB171 strains harboring either the transcriptional fusion plasmid pHT1K-*P_prpD_*-*lacZ*, pHT1K-*P_prp_*-*lacZ*, or the empty vector control pHT1K-*lacZ* was cultured at 28 °C in 200 mL GYS medium supplemented with 25 μg/mL erythromycin, with or without propionate. 4 mL culture aliquots were collected every 2 h for β-galactosidase activity assay, expressed in Miller units, as described previously (Zheng et al., 2015).

### Identification of transcription start site (TSS)

The TSS of the *prpD* gene was identified using a 5′ rapid amplification of cDNA ends (5′-RACE) method. Total RNA was extracted from BMB171 cultured in GYS medium for 12 h. First-strand cDNA was synthesized using a PrimeScript RT reagent Kit with gDNA Eraser (Takara, Japan). The cDNA was purified using a Nucleic Acid Purification Kit (Axygen, China). A poly(A) tail was added to the 3′ end of the purified cDNA using terminal deoxynucleotidyl transferase (TdT). The tailed cDNA was then used as a template for PCR amplification with a 5′-RACE adaptor primer containing a poly(T) sequence and a nested *prpD*-specific primer. The PCR product was purified, sequenced, and the nucleotide immediately adjacent to the 5’-RACE adaptor sequence was identified as the TSS.

### Statistical analysis

One-way ANOVA and Tukey’s honest significant difference test was used to evaluate the differences in promoter (*P_prpD_* and *P_prp_*) activity at different propionate concentrations. To evaluate the overall difference in growth capacity among different *B. thuringiensis* strains, the area under each curve (AUC) was calculated as a comprehensive growth index. Subsequently, the Mann–Whitney U test was used to statistically compare the AUC values between the two groups. IBM SPSS (Statistical Package for the Social Sciences) software (Version 20.0) was used for these analyses. Statistical significance is indicated by asterisks (**p < 0.01, ****p < 0.0001).

## Results

### 2-Methylcitrate is a toxic metabolite of *B. thuringiensis*

**Figure 2:**
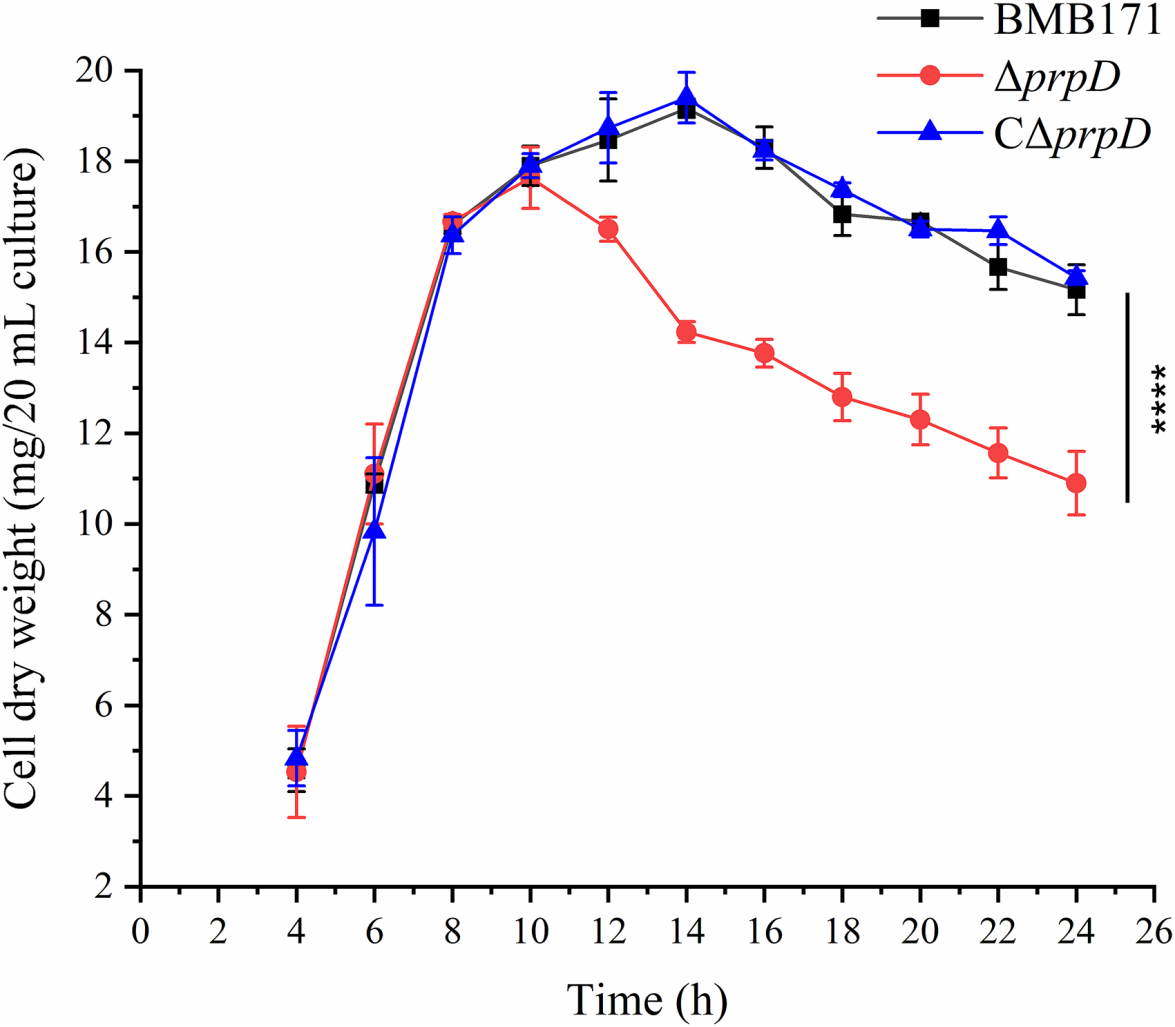
Deletion of the *prpD* inhibits the growth of *B. thuringiensis* and reduces cell biomass. The parental-type BMB171, the Δ*prpD* mutant, and the complementary strain CΔ*prpD* were cultured in GYS medium at 28 °C. Cell dry weight was measured at specified time points. The data showed that the biomass of the Δ*prpD* strain decreased sharply after entering the stationary phase. All data are expressed as mean ± standard error of three independent biological replicates.

The *prpD* gene encodes 2-methylcitrate dehydratase, which converts 2-methylcitrate into 2-methyl-*cis*-aconitate. Our previous work showed that while 2-methylcitrate synthesis in *B. thuringiensis* BMB171 is restricted to the stationary phase, the deletion of *prpD* leads to a significant accumulation of this metabolite compare to the parental strain BMB171 (Zheng et al., 2020). To investigate whether this accumulation is harmful to *B. thuringiensis*, we compared the growth profiles of parental-type BMB171 and Δ*prpD* mutant. As shown in Figure 2, during the exponential growth phase, the cell dry weights of the two strains were comparable. However, after entering the stationary phase (approximately 10 h), the cell dry weight of the Δ*prpD* strain decreased sharply, whereas that of BMB171 remained relatively stable, consistent with the normal bacterial growth cycle. The cell dry weight of the complementary strain CΔ*prpD* is not significantly different from that of the parental strain BMB171 (Figure 2). This result confirms that deletion of the *prpD* gene has an adverse effect on the growth of *B. thuringiensis*. This effect can be primarily attributed to the toxic accumulation of 2-methylcitrate during the stationary phase.

**Figure 3:**
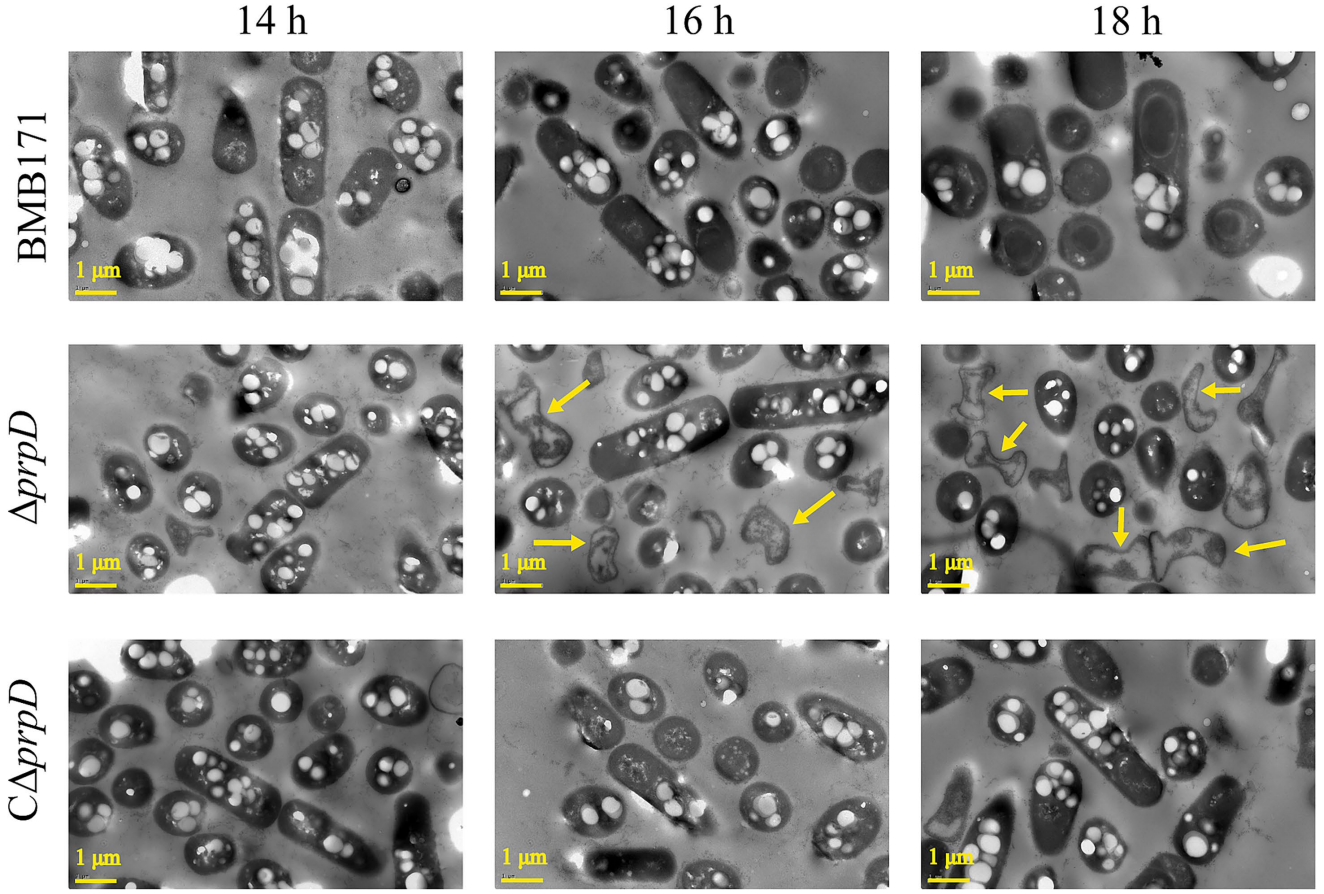
Accumulation of 2-methylcitrate induces lysis in *B. thuringiensis* cells. Strains BMB171, Δ*prpD*, and CΔ*prpD* were cultured in GYS medium at 28 °C. Cells were collected at specified time points (14 h, 16 h, 18 h) and observed using transmission electron microscopy (TEM). The number of lysed cells in the Δ*prpD* culture gradually increased over time. Yellow arrows in the figure indicate representative lysed cells. Scale bars, 1 μm.

To further investigate cellular effects, we observed the morphology of BMB171, Δ*prpD* and CΔ*prpD* cells using transmission electron microscopy. After 14 to 18 h of cultivation, an increasing number of Δ*prpD* cells showed signs of lysis and death (Figure 3). In contrast, even at 18 h, only a small number of lysed cells were observed in the cultures of BMB171 and the complementary strain CΔ*prpD* (Figure 3). Taken together, these macroscopic and microscopic observations indicate that 2-methylcitrate is a toxic metabolite that has a severe adverse impact on the viability of *B. thuringiensis*, especially during the stationary phase.

### Accumulation of 2-methylcitrate leads to overall metabolic imbalance in *B. thuringiensis*

To elucidate the molecular mechanisms of 2-methylcitrate toxicity, we conducted comparative transcriptomic analysis (RNA-seq) on strains BMB171 and Δ*prpD*. Samples were collected at 12 h, at which point significant differences in biomass began to appear. Compared to the parental-type, 3,036 differentially expressed genes (DEGs) were identified in strain Δ*prpD*, including 1,539 upregulated genes and 1,497 downregulated genes.

**Figure 4:**
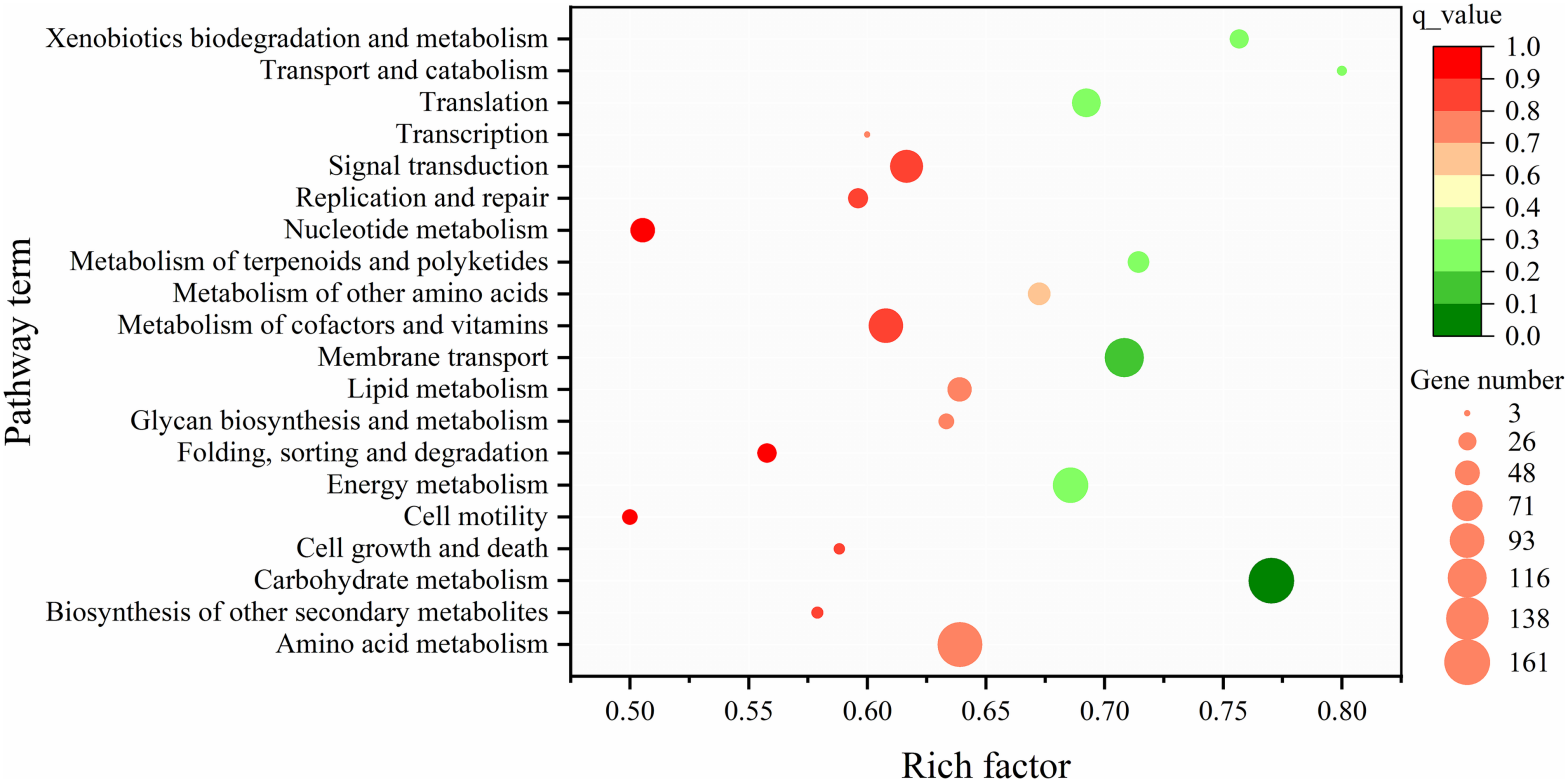
KEGG pathway enrichment analysis of differentially expressed genes (DEGs). This analysis was based on DEGs identified between Δ*prpD* and BMB171. Dots in the figure represent pathway enrichment, with different colors representing different *q*_values. Green indicates high enrichment, and red indicates low enrichment. The size of the dot is proportional to the number of DEGs enriched in that pathway.

Pathway enrichment analysis using the Kyoto Encyclopedia of Genes and Genomes (KEGG) revealed that the most significantly affected pathway was “carbohydrate metabolism” (*q*_value = 2.5 × 10^−5^) (Figure 4). Glycolysis pathway and the TCA cycle are two of the most important and sequentially linked pathways in central carbon metabolism. Therefore, we focused our analysis on DEGs within these pathways. The results showed that, exception for the glucokinase gene, the transcripts of all other enzymes in the glycolysis pathway were significantly downregulated in the Δ*prpD* strain. Notably, the gene encoding glyceraldehyde-3-phosphate dehydrogenase was downregulated most significantly, decreasing by nearly 300-fold (Table 2). This indicates a significantly slower rate of glycolysis in the Δ*prpD* mutant.

**Table 2:** Comparative analysis of the transcription profiles of the genes in glycolysis pathway and TCA cycle between BMB171 and ∆*prpD.*

Gene code	Gene name	Gene function	RPKM				log_2_(Fold_change) normalized (∆*prpD vs* BMB171)	*q*_value	Change (∆*prpD vs* BMB171)
			∆*prpD*-1	∆*prpD*-2	171–1	171–2			
Glycolysis pathway									
BMB171_RS21265	*glcK*	Glucokinase	98.4	80.9	131.9	125.8	−0.54	5.59E-10	Unchanged
BMB171_RS24450	*pgi*	Glucose-6-phosphate isomerase	110.9	90.8	1127.4	961.2	−3.39	0	Down
BMB171_RS22885	*pfkA*	6-phosphofructokinase	85.4	96.3	490.7	379.4	−2.24	7.01E-272	Down
BMB171_RS26655	*fba2*	Fructose-bisphosphate aldolase	670.0	524.6	4427.8	4153.1	−2.87	0	Down
BMB171_RS25575	*tpiA*	Triosephosphate isomerase	123.1	84.9	3158.2	3167.9	−4.96	0	Down
BMB171_RS25585	*gap2*	Glyceraldehyde-3-phosphate dehydrogenase	8.9	8.7	2274.2	2959.6	−8.22	0	Down
BMB171_RS25580	*pgk*	Phosphoglycerate kinase	109.1	106.9	2393.6	2345.2	−4.46	0	Down
BMB171_RS25570	*gpmI*	Phosphoglyceromutase	79.9	89.2	2217.7	2233.6	−4.71	0	Down
BMB171_RS25565	*eno*	Enolase	46.8	53.5	1638.1	1797.2	−5.09	0	Down
BMB171_RS22880	*pyk*	Pyruvate kinase	221.3	154.1	1360.3	1385.0	−2.90	0	Down
TCA cycle									
BMB171_RS19700	*pdhB*	Pyruvate dehydrogenase E1 component subunit beta	63.3	71.0	1541.2	2580.7	−4.94	0	Down
BMB171_RS19705	*pdhA*	Pyruvate dehydrogenase E1 component subunit alpha	82.6	64.7	1274.1	2528.6	−4.73	0	Dwon
BMB171_RS19695	*pdhC*	Branched-chain alpha-keto acid dehydrogenase subunit E2	153.0	120.7	4197.3	5358.6	−5.15	0	Down
BMB171_RS19690	*pdhD*	Dihydrolipoamide dehydrogenase	44.6	37.8	1513.4	1911.6	−5.40	0	Down
BMB171_RS22855	*citZ*	Citrate synthase	1262.1	1602.1	111.9	116.7	3.67	0	Up
BMB171_RS17915	*acnA*	Aconitate hydratase	20944.6	17339.4	258.5	221.4	6.30	0	Up
BMB171_RS22850	*citC*	Isocitrate dehydrogenase	2192.3	2102.4	51.6	34.9	5.64	0	Up
BMB171_RS06375	*odhB*	Dihydrolipoamide acetyltransferase	154.9	225.0	50.0	49.3	1.97	2.41E-136	Up
BMB171_RS18705	*korA*	2-oxoglutarate ferredoxin oxidoreductase subunit alpha	1294.3	1124.2	425.2	348.3	1.64	0	Up
BMB171_RS18700	*korB*	2-oxoglutarate ferredoxin oxidoreductase subunit beta	755.4	902.4	392.9	294.3	1.29	1.80E-219	Up
BMB171_RS19005	*sucD*	Succinyl-CoA synthetase alpha subunit	3341.9	1963.9	97.2	87.0	4.80	0	Up
BMB171_RS19010	*sucC*	Succinyl-CoA synthetase beta subunit	2159.8	1629.5	70.3	35.9	5.14	0	Up
BMB171_RS22465	*sdhA*	Succinate dehydrogenase flavoprotein subunit	1201.2	757.1	87.9	61.1	3.68	0	Up
BMB171_RS22460	*sdhB*	Succinate dehydrogenase iron–sulfur subunit	826.6	665.9	60.8	60.0	3.61	0	Up
BMB171_RS22470	*sdhC*	Succinate dehydrogenase cytochrome b556 subunit	632.8	381.7	71.0	43.0	3.12	2.57E-286	Up
BMB171_RS08790	*fumC*	Fumarate hydratase, class II	2692.2	1783.1	221.7	164.3	3.50	0	Up
BMB171_RS22845	*mdh*	Malate dehydrogenase	1373.9	1147.6	59.3	48.7	4.53	0	Up

Given that the cultures were grown under aerobic conditions, pyruvate, the final product of glycolysis, was expected to be further oxidized via the TCA cycle. However, in the Δ*prpD* strain, the transcriptional profile of the TCA cycle was opposite to that of glycolysis. Except for the repression of the pyruvate dehydrogenase complex genes, the transcripts of all TCA cycle enzymes were significantly upregulated. The most significantly upregulated gene was aconitase, with a 79-fold increase in expression (Table 2). Such a significant transcriptomic difference between these two consecutive and tightly coupled pathways suggests a severe metabolic disorder in the Δ*prpD* mutant. This metabolic imbalance, the inhibition of glycolysis and the activation of TCA cycle is a key factor contributing to the observed cell lysis and death.

### *B. thuringiensis* responds to 2-methylcitrate toxicity via an internal promoter

**Figure 5:**
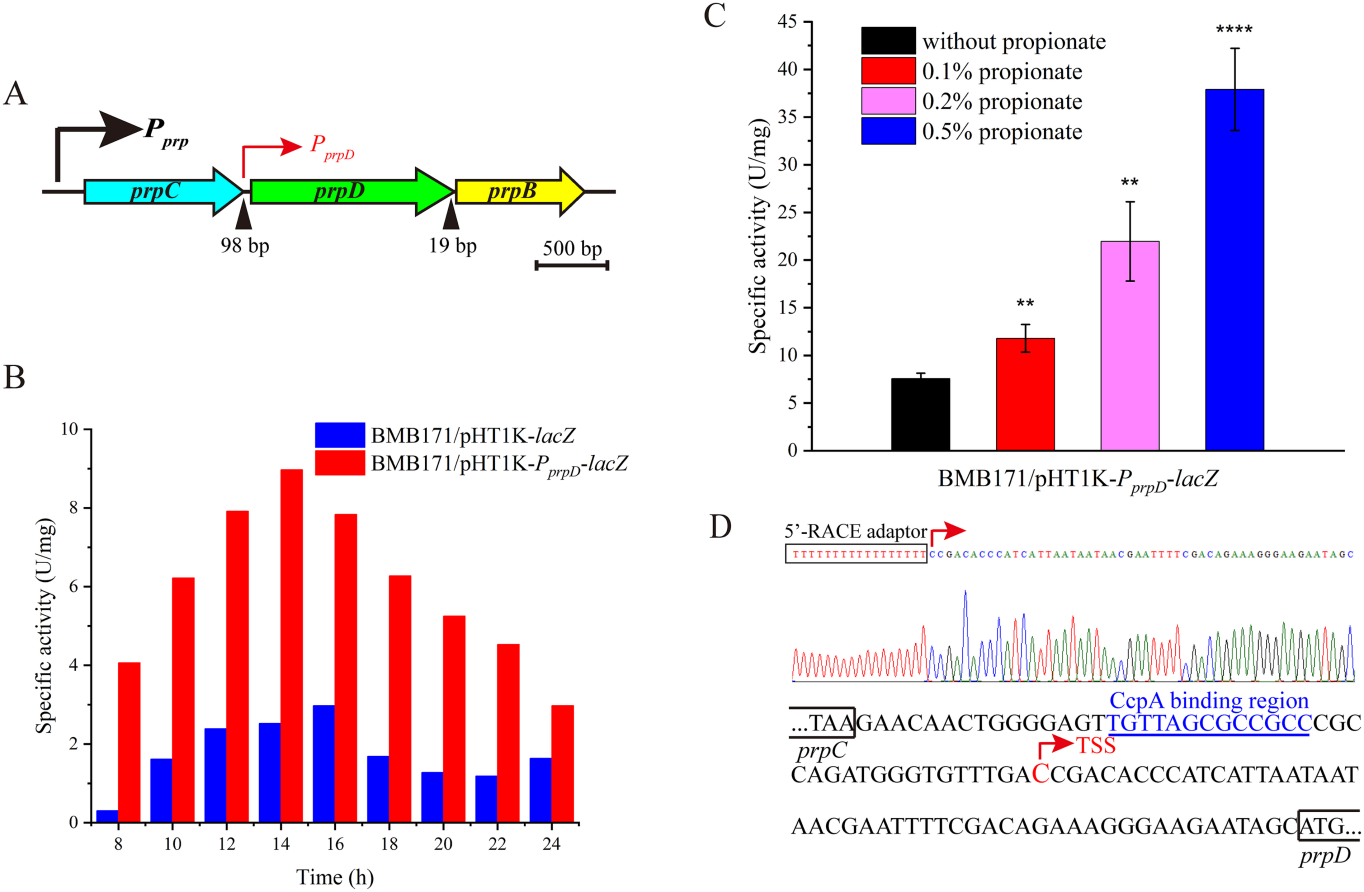
Identification and characterization of the independent internal promoter within the *B. thuringiensis prp* operon. **(A)** Schematic diagram of the *prp* operon architecture. This operon contains a major promoter (*P_prp_*) that controls the entire operon and an independent internal promoter (*P_prpD_*) located upstream of the *prpD* gene. **(B)** *β*-galactosidase activity assay confirmed the function of this internal promoter. The activity of *P_prpD_* was measured using the reporter strain BMB171/pHT1K-*P_prpD_*-*lacZ*. The strain BMB171/pHT1K-*lacZ* carrying the empty vector served as a negative control. **(C)** Propionate induced the activity of this internal promoter in a dose-dependent upregulation. The strain was cultured in GYS medium with or without propionate at 28 °C for 18 h to measure β-galactosidase activity. All data are expressed as mean ± standard error of three independent biological replicates. **(D)** Transcriptional start site (TSS) of the internal promoter was located using 5′-RACE technology. The identified TSS, shown in red, is located 52 bp upstream of the *prpD* start codon. The region marked in blue is the potential CcpA-binding site. **p < 0.01, ****p < 0.0001.

Our previous transcriptomic data from *B. thuringiensis* CT-43 showed that at 7 and 9 h, the RPKM value of the *prpD* gene was 3–4 times higher than that of the first gene *prpC* in the *prp* operon (Wang et al., 2013). This suggests the possible existence of an independent promoter upstream of the *prpD* gene (Figure 5A).

To test this hypothesis, we cloned a 250 bp fragment from the upstream region of the *prpD* gene into the *lacZ* reporter vector pHT1K-*lacZ*, constructing the pHT1K-*P_prpD_*-*lacZ* plasmid. This plasmid was transformed into strain BMB171, and the change in *β*-galactosidase activity over time was monitored. As shown in Figure 5B, the strain carrying pHT1K-*P_prpD_*-*lacZ* showed a significant increase in β-galactosidase activity from 8 to 14 h, peaking at 14 h, and then gradually declining. In contrast, the control strain carrying the empty vector showed almost no activity. Furthermore, when 0.1%, 0.2%, and 0.5% propionate were added to the culture medium, the activity of this promoter was significantly enhanced in a stepwise manner, indicating that its expression level increases progressively with rising propionate concentration and exhibits a clear dose-dependent induction pattern (Figure 5C).

We further located the transcription start site (TSS) of this promoter using 5′-RACE technology. The TSS was identified to locate 52 bp upstream of the *prpD* start codon (Figure 5D). The presence of this independent promoter enhances PrpD expression, and is unaffected by the main operon promoter. This regulatory mechanism may be a detoxification strategy, enabling cells to rapidly synthesize more PrpD, thereby degrading accumulated 2-methylcitrate and mitigating its toxic effects.

## Discussion

The 2-methylcitrate cycle is a double-edged sword for microorganisms (Dolan et al., 2018). While it provides a pathway for microorganisms to catabolize propionic acid for energy and biomass, it also produces toxic intermediates, such as 2-methylcitrate. In this study, we showed that 2-methylcitrate has a significant inhibitory effect on the growth of *B. thuringiensis*. This was manifested at both macroscopic and microscopic levels: macroscopically, it is characterized by a sharp decrease in the dry weight of Δ*prpD* mutant cells during the stationary phase; microscopically, it is characterized by increased cell lysis and death observed under transmission electron microscopy. These findings clearly confirm the toxicity of 2-methylcitrate to *B. thuringiensis*. Notably, however, a mutant lacking the entire 2-methylcitrate cycle (Δ*prpCDB*) exhibited no significant growth difference compared to the parental strain BMB171 in GYS medium (Supplementary Figure S1). We previously determined the 2-methylcitrate content of strains BMB171, Δ*prpD*, and Δ*prpCDB* at different growth stages (6 h in logarithmic growth phase; 12 h in early stationary phase) (Zheng et al., 2020). Changes in 2-methylcitrate content were consistent with the specific expression of the *prp* operon during the stationary phase and the function of related genes within the *prp* operon. We believe that the growth differences of strains BMB171, Δ*prpD*, and Δ*prpCDB* may be related to the accumulation of 2-methylcitrate: in BMB171, although 2-methylcitrate is present, it is metabolized by PrpD in a timely manner, resulting in a low concentration that does not affect growth; however, in Δ*prpCDB* mutant, the metabolic cycle is completely blocked, preventing the formation of 2-methylcitrate and thus preventing toxic accumulation; while in strain Δ*prpD*, 2-methylcitrate accumulates in large quantities and cannot be further metabolized, leading to significant growth inhibition. Even with only pure glucose added to the GYS medium and no pure propionic acid carbon source, the *prp* operon was still transcribed normally in GYS medium. This indicates that the 2-methylcitrate cycle functions normally in GYS medium. This may be because propionyl-CoA produced by *B. thuringiensis*’ own metabolism (odd fatty acid degradation and amino acid metabolism, etc.) or potential propionic acid carbon source in yeast extracts require both metabolism and utilization through the 2-methylcitrate cycle. Based on this, we believe that as long as the 2-methylcitrate cycle functions normally, the accumulation of 2-methylcitrate in the *prpD* deletion mutation does not necessarily require the presence of an additional propionic acid carbon source in the culture medium. Notably, the formation and accumulation of 2-methylcitrate may occur not only through the canonical 2-methylcitrate cycle but also via a potential mechanism involving the reverse methylcitrate cycle (Serafini et al., 2019).

We also considered an alternative metabolic route, as some bacteria utilize the vitamin B_12_-dependent methylmalonyl pathway to metabolize propionate or propionyl-CoA (Savvi et al., 2008). To test this possibility, we supplemented the GYS medium with various concentrations of vitamin B_12_ (10–50 μg/mL), but this failed to rescue the cytotoxic phenotype of the Δ*prpD* mutant (Supplementary Figure S2). Consistent with this experimental result, our bioinformatic analysis did not identify a gene encoding methylmalonyl-CoA mutase, the key signature enzyme of this pathway, in the *B. thuringiensis* BMB171 genome. Therefore, it is unlikely that *B. thuringiensis* BMB171 possesses a functional methylmalonyl pathway.

Our comparative transcriptomic analysis provides a mechanistic explanation for this toxicity. Accumulation of 2-methylcitrate triggered a global transcriptional reprogramming in *B. thuringiensis*, affecting over 3,000 genes, indicating a widespread cellular stress response. The most striking finding was a severe imbalance in central carbon metabolism. The consistent downregulation of glycolysis pathway genes and the simultaneous upregulation of TCA cycle genes represent a complete decoupling of these two fundamental and interconnected pathways. For efficient energy production, the transcriptional activity of these pathways must be coordinated. In the Δ*prpD* strain, this loss of coordination, leads to severe metabolic conflict, ultimately resulting in cell death. Consistent with our transcriptomic data, we also observed a significant accumulation of pyruvate in the Δ*prpD* strain (data not shown), further supporting the conclusion that the metabolic flux from glycolysis to the TCA cycle is disrupted. This finding not only explains its toxic phenotype but also provides potential avenues for metabolic engineering, such as using this genetic background to overproduce pyruvate.

The exact molecular mechanisms by which 2-methylcitrate exerts its toxic effects is not fully elucidated. Several possibilities exist: (1) as a small organic acid, its accumulation can lower intracellular pH, thereby disrupting various cellular processes; (2) it may act as a competitive or allosteric inhibitor of key metabolic enzymes, directly interfering with metabolic flux; (3) it may act as a signaling molecule, interacting with specific transcription factors to regulate the expression of downstream genes; or (4) the detrimental effects may arise from other metabolic perturbations rather than 2-methylcitrate itself. These hypotheses all warrant further investigation. Regardless of the specific mechanism, the critical role of PrpD in detoxifying 2-methylcitrate makes it an attractive potential target for developing novel anti-bacterial drugs.

Bacteria have evolved various strategies to cope with the accumulation of toxic metabolic intermediates. For example, in *Pseudomonas aeruginosa*, the citrate lyase AceA exhibits secondary 2-methylisocitrate lyase activity, which can serve as an alternative to mitigate toxicity when the major enzyme PrpB is non-functional (Wijaya et al., 2025). In this study, we discovered a different strategy in *B. thuringiensis*: an additional, independent promoter exists within the *prp* operon. Compared to the “enzyme redundancy” strategy reported in *P. aeruginosa*, the detoxification mechanism of *B. thuringiensis* seems to exhibit some evolutionary advantage. For example, it does not simply add a functional backup for detoxification, but rather by constructs a more complex gene regulatory network, thereby enabling faster, more economical, and more robust initiation of a stress response. This strategy allows cells to respond efficiently to lethal metabolic stress with minimal cost. By analyzing the DBTBS database (Sierro et al., 2008), we discovered that the potential binding sites of a global transcription factor CcpA (the carbon catabolite protein A) is situated upstream of the *prpD* gene. Consistent with the observed trend of progressively increasing activity from the logarithmic to the stationary phase (Figure 5B), we hypothesize that CcpA may repress the internal promoter during the exponential phase, an inhibition that is subsequently relieved upon entry into the stationary phase. This regulatory mechanism is congruent with CcpA-mediated carbon catabolite repression (CCR) commonly observed in Gram-positive bacteria. However, given the significant diversity in the organization and gene composition of 2-methylcitrate cycle operons across different bacterial species (Zheng et al., 2020), whether such independent internal promoters are a conserved feature remains to be further elucidated. Notably, although the major promoter was significantly more active than the inner promoter, both the internal and primary promoters exhibit a distinct propionate-dependent induction pattern (Figure 5C; Supplementary Figure S3), suggesting that the *prp* operon can rapidly sense fluctuations in environmental propionate or its metabolic intermediates. Such a mechanism enables the cell to respond to nutritional shifts in a more parsimonious and efficient manner. Overall, this internal promoter drives elevated expression of *prpD* gene (and possibly downstream *prpB* gene), thereby promoting rapid and efficient clearance of toxic 2-methylcitrate. This represents a sophisticated regulatory mechanism designed to address the inherent risks associated with propionic acid metabolism.

## Data Availability

The data presented in this study are publicly available. The data can be found at: https://www.ncbi.nlm.nih.gov/geo, accession GSE140317.
