# Supplementary material for: Accumulation of 2-methylcitrate induces metabolic imbalance in Bacillus thuringiensis, revealing a detoxification strategy mediated by an internal promoter
Source: Front Microbiol. 2026 Jan 28;17:1675856. doi: 10.3389/fmicb.2026.1675856 (PMC12891235; doi:10.3389/fmicb.2026.1675856)
Supplement: Supplementary file 1 [file Table_1.DOC]

**Table S1.** **Primers used in this study**

| **Primers** | **Primer sequences (5'-3')** | **Use** |
| --- | --- | --- |
| RACE-adaptor-F | GACCACGCGTATCGATGTCGACTTTTTTTTTTTTTTTT | 5′-RACE experiment |
| RACE-*prpD*-R | taaaattccgcctaggttatcagatgg |
| *P_prpD_*-*lacZ*-F | CCATGGATGCTAGGTATTCCAATTCAAC | Amplification of *P_prpD_* for β-galactosidase assay |
| *P_prpD_*-*lacZ*-R | GGATCCGCTATTCTTCCCTTTCTGTCG |
| C*prpD* F | GTCGACAAGAAGAATATGCAGTACC | Construction of the *prpD* complementary vector pRP1028-C*prpD* |
| C*prpD* R | GGTACCGCTTTCATAGCTTTTTGCT |
