# Supplementary material for: Accumulation of 2-methylcitrate induces metabolic imbalance in Bacillus thuringiensis, revealing a detoxification strategy mediated by an internal promoter
Source: Front Microbiol. 2026 Jan 28;17:1675856. doi: 10.3389/fmicb.2026.1675856 (PMC12891235; doi:10.3389/fmicb.2026.1675856)
Supplement: Supplementary file 2 [file Image_1.PDF]

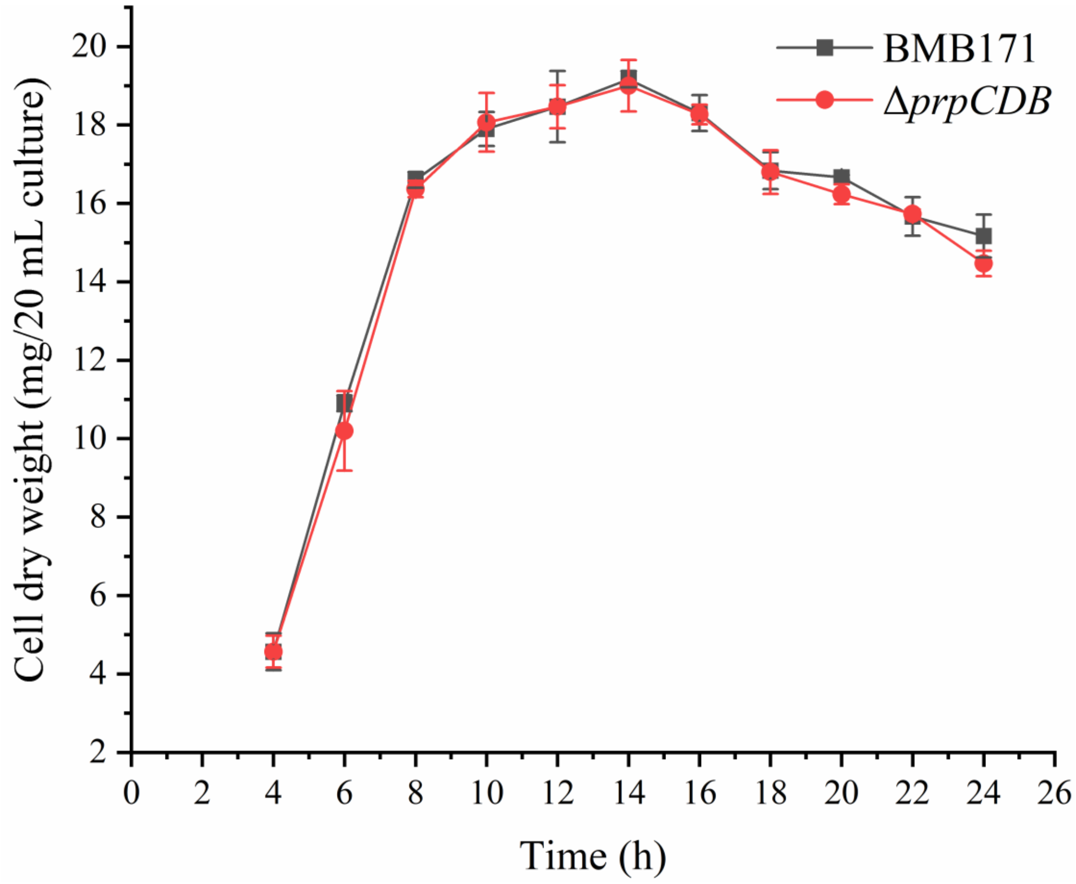

Figure S1. Detection the growth curves of BMB171 and Δ*prpCDB*. All of the strains were cultured in GYS medium at 28°C. Cell dry weight was measured at specified time points. No significant difference was observed in the biomass among the three strains ($P > 0.05$). All data are expressed as mean ± standard error of three independent biological replicates.

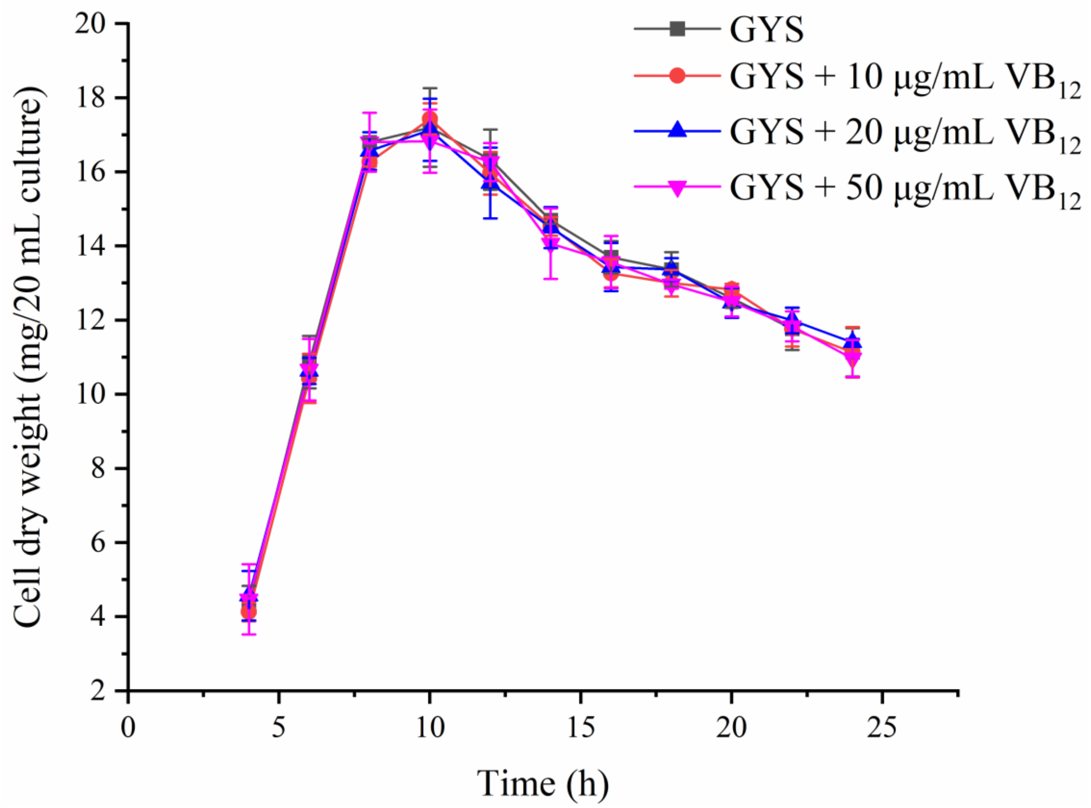

Figure S2. Effect of different vitamin B_{12} concentrations on the growth of the Δ*prpD* mutant. Δ*prpD* mutant with different vitamin B_{12} concentrations were cultured in GYS medium at 28°C. Cell dry weight was measured at specified time points. Supplementation with vitamin B_{12} (10-50 μg/mL) did not significantly affect the growth of the Δ*prpD* mutant ($P > 0.05$). All data are expressed as mean ± standard error of three independent biological replicates.

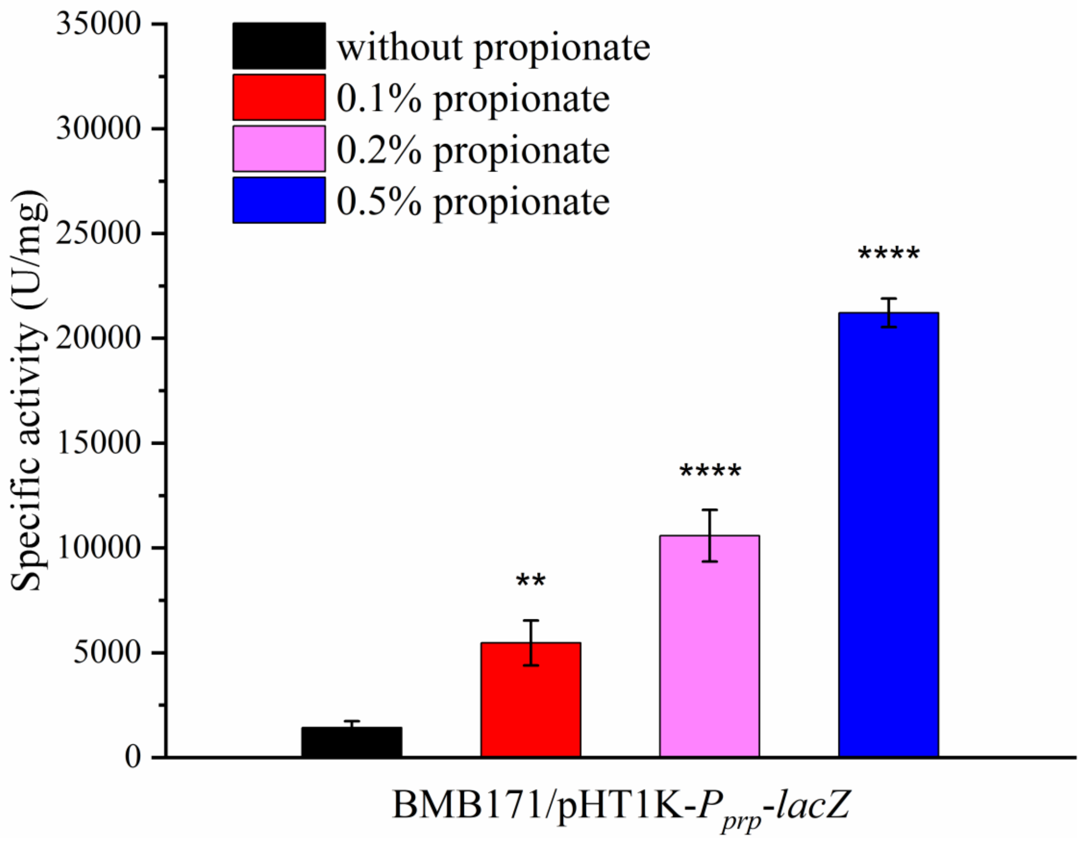

Figure S3. The activity of the major promoter of the *prp* operon was also induced by propionate, exhibiting a dose-dependent upregulation. Strains were cultured in GYS medium with or without propionate at 28°C for 18 hours to determine the β-galactosidase activity. All data are expressed as mean ± standard error of three independent biological replicates.
